# Engineering cofacial porphyrin dimers using lacunary polyoxotungstates†

**DOI:** 10.1039/d5sc00814j

**Published:** 2025-04-22

**Authors:** Masahiro Yamaguchi, Kentaro Yonesato, Kaito Shioya, Chifeng Li, Kei Murata, Kazuyuki Ishii, Kazuya Yamaguchi, Kosuke Suzuki

**Affiliations:** a Department of Applied Chemistry, School of Engineering, The University of Tokyo 7-3-1 Hongo, Bunkyo-ku Tokyo 113-8656 Japan ksuzuki@appchem.t.u-tokyo.ac.jp kyama@appchem.t.u-tokyo.ac.jp; b Institute of Industrial Science, The University of Tokyo 4-6-1 Komaba, Meguro-ku Tokyo 153-8505 Japan k-ishii@iis.u-tokyo.ac.jp; c RIKEN Center for Sustainable Resource Science 2-1 Hirosawa, Wako-shi Saitama 351-0198 Japan

## Abstract

Cofacial porphyrin dimers have garnered extensive attention for their unique photophysical and catalytic properties, which strongly depend on structural configurations. However, precisely controlling key parameters, such as lateral and rotational displacements, interfacial distance, and stability, remains challenging. Herein, we present a novel strategy for engineering porphyrin dimer structures and properties using multivacant lacunary polyoxometalates (POMs), [SiW_10_O_36_]^8−^ or [SiW_9_O_34_]^10−^, as linkers. By adjusting the types and coordination modes of lacunary POMs, three distinct hybrids were obtained *via* the self-assembly of two 5,10,15,20-tetra(4-pyridyl)porphyrin molecules and four lacunary POM units, each exhibiting modulated stacking geometries, interfacial distances and interactions, and photophysical properties. These hybrids demonstrated efficient visible-light-responsive photosensitized reactions to generate singlet oxygen 
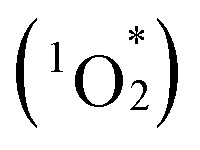
 from ground-state triplet oxygen (^3^O_2_), leading to the photooxidation of various organic substrates. Notably, hybrid II, constructed using [SiW_10_O_36_]^8−^, exhibited the strongest π–π interactions, distinct optical properties, and enhanced resistance to 
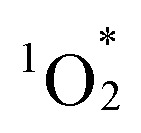
-induced degradation. These findings highlight the potential of POMs as versatile tools for the precise control of porphyrin dimer architectures and the development of materials with tailored photophysical and catalytic functions.

## Abstract 2

This work reports a novel strategy for engineering porphyrin dimer structures and properties using lacunary polyoxometalates. Three distinct hybrids exhibit modulated stacking geometries, interfacial distances, and properties.

## Graphical abstract

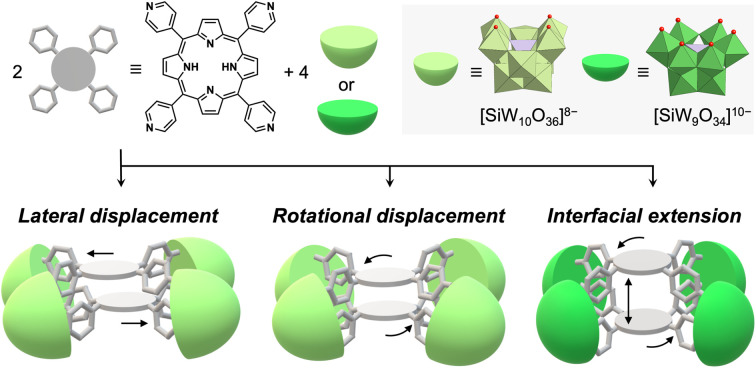

## Introduction

Cofacial porphyrin dimers, characterized by two porphyrins arranged in a face-to-face configuration through linkers, have attracted considerable attention owing to their unique photophysical and catalytic properties. These dimers function as minimal models for photosynthetic reaction centers,^1^ demonstrate photophysical characteristics,^2^ exhibit catalytic activities,^3^ and facilitate host–guest interactions,^4^ all dictated by their specific structural configurations. Consequently, precise control over the spatial arrangement of porphyrins within these dimers—specifically lateral and rotational displacements as well as interfacial distance—is crucial, as these structural parameters directly influence their photophysical and catalytic properties.^5^ Although various strategies for synthesizing cofacial porphyrin dimers have been explored to date, including the use of organic linkers,^6^ metal-ion linkers,^7^ and supramolecular interactions,^8^ challenges persist in achieving precise structural control and in addressing stability issues.

**Fig. 1:**
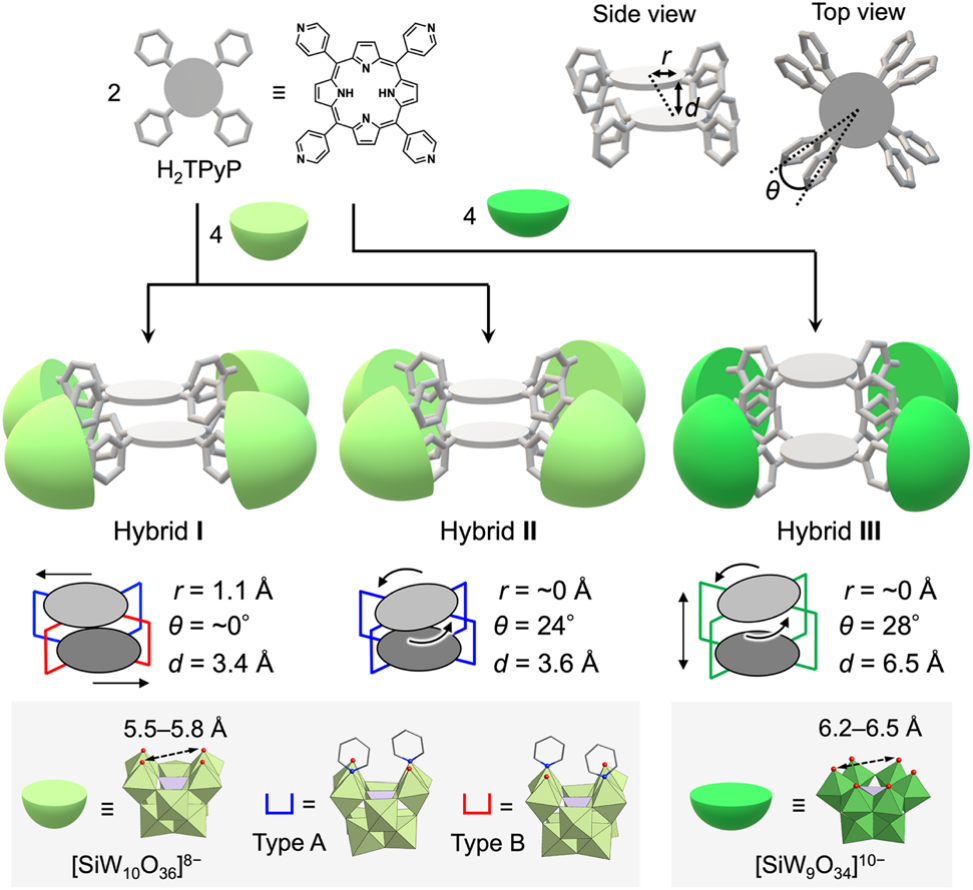
Schematic depiction of the self-assembly process for engineering cofacial porphyrin dimers (hybrids I, II, and III) with distinct lateral displacement (*r*), rotational displacement (*θ*), and interfacial distance (*d*) by using H_2_TPyP and multivacant lacunary polyoxotungstates ([SiW_10_O_36_]^8−^ or [SiW_9_O_34_]^10−^).

To address these challenges, we propose the use of polyoxometalates (POMs),^9^ anionic metal oxide clusters, as linkers. In particular, multivacant lacunary POMs (*e.g.*, [XM_10_O_36_]^*n*−^ and [XM_9_O_34_]^*n*−^, where X = Si, P, Ge, or As, and M = Mo or W) are generated by removing several {MO_*x*_} units from their parent structures ([XM_12_O_40_]^*n*−^). They provide highly reactive vacant sites that can coordinate with metal ions^10^ or organic ligands,^11,12^ facilitating the construction of versatile functional materials. Recently, we reported the self-assembly of a porphyrin–POM hybrid photocatalyst (hybrid I), comprising a stacked 5,10,15,20-tetra(4-pyridyl)porphyrin (H_2_TPyP) dimer bridged by four divacant lacunary polyoxotungstates ([SiW_10_O_36_]^8−^) through the coordination of pyridyl groups in H_2_TPyP to W atoms at the vacant sites.^13^ Hybrid I featured a laterally displaced porphyrin dimer structure without rotational displacement (Fig. 1).

We herein propose that lacunary POMs offer three primary advantages for constructing porphyrin dimers. First, their rigid frameworks stabilize the stacked porphyrin arrangement, preventing degradation. Second, the diverse coordination modes of lacunary POMs enable fine-tuning of porphyrin displacement. For instance, [SiW_10_O_36_]^8−^ provides two enantiomeric coordination modes for ligands (types A and B, Fig. 1). In hybrid I, the four {SiW_10_} units exhibited two type A and two type B configurations. Third, the inter-porphyrin distance can be adjusted by selecting different POMs, such as divacant [SiW_10_O_36_]^8−^ (5.5–5.8 Å; distance between O atoms at the vacant sites) and trivacant [SiW_9_O_34_]^10−^ (6.2–6.5 Å).

In this study, building on these proposed advantages, we present a novel method for constructing cofacial porphyrin dimers with precisely controlled stacked structures using multivacant lacunary POMs as linkers (Fig. 1). This approach grants control over key structural parameters governing porphyrin stacking, including lateral displacement (*r*), rotational displacement (*θ*), and interfacial distance (*d*), providing a unique platform for fine-tuning photophysical and catalytic properties. Adopting this strategy, we synthesized and characterized new porphyrin dimers, hybrids II and III, each featuring distinct stacking arrangements compared to the previously reported hybrid I. Their photophysical and photocatalytic properties were systematically investigated. Hybrid II was formed through the self-assembly of two H_2_TPyP molecules and four [SiW_10_O_36_]^8−^ units under reaction conditions different from those for I, yielding a cofacial porphyrin dimer with no lateral displacement but with rotational displacement. In contrast, hybrid III featured an extended inter-porphyrin distance, achieved by replacing [SiW_10_O_36_]^8−^ with a trivacant lacunary polyoxotungstate, [SiW_9_O_34_]^10−^, further diversifying the stacking configuration. Notably, hybrid II displayed the strongest π–π interactions between porphyrins in all three hybrids owing to its tightly stacked configuration, which induced pronounced changes in its optical properties, including ultraviolet-visible (UV-vis) absorption, fluorescence, and phosphorescence spectra. These boosted π–π interactions also conferred superior resistance against 
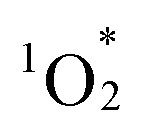
-induced degradation, a common limitation of porphyrins. These findings underscore the versatility of lacunary POMs as molecular linkers for developing cofacial porphyrin dimers with precisely tunable structural parameters. By granting control over lateral displacement, rotational displacement, and interfacial distance, the proposed approach offers a powerful tool for the rational design of advanced materials with tailored photophysical and catalytic functionalities.

## Results and discussion

### Synthesis and characterization

**Fig. 2:**
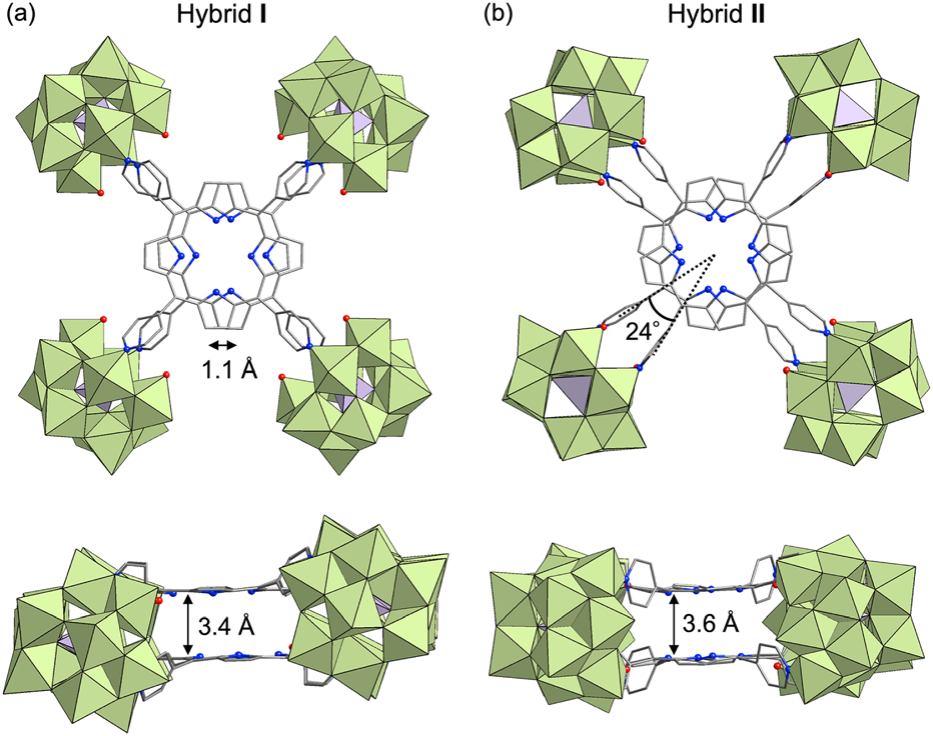
Crystal structures of the anionic components of (a) hybrid I and (b) hybrid II (top and side views). Color code: W, light green; Si, purple; C, gray; N, blue; O, red. H atoms are omitted for clarity.

In a recent study, we successfully synthesized hybrid I, featuring two cofacially stacked porphyrins stabilized through coordination with four divacant lacunary [SiW_10_O_36_]^8−^ units (Fig. 2a).^13^ This hybrid was obtained through a self-assembly process by reacting TBA_4_H_4_[SiW_10_O_36_] (TBA = tetra-*n*-butylammonium) and H_2_TPyP in a 2 : 1 molar ratio in a mixture of *N*,*N*-dimethylacetamide (DMA) and 1,2-dichloroethane (DCE) at 80 °C. Hybrid I displayed a Soret band at 406 nm, which was considerably blue-shifted compared to that of 5,10,15,20-tetraphenylporphyrin (H_2_TPP, 413 nm) owing to its stacked dimer structure. Although hybrid I featured two distinct coordination modes of {SiW_10_} units (types A and B, Fig. 1), we hypothesized the existence of other stable configurations involving a single coordination mode. To explore this possibility, we performed the reaction in various solvent systems. When TBA_4_H_4_[SiW_10_O_36_] and H_2_TPyP were reacted in solvents, such as DMA, acetonitrile, and nitromethane at 80 °C, the UV-vis spectra of the resulting reaction mixtures consistently displayed Soret bands at 405–406 nm, closely resembling the Soret band of hybrid I, synthesized in a mixture of DMA/DCE (Fig. S1 and Table S1†). However, when the reaction was performed in *N*,*N*-dimethylformamide (DMF), the UV-vis spectrum displayed a Soret band at 399 nm, which was significantly blue-shifted compared to that of hybrid I (Fig. S1 and Table S1†). This shift strongly suggests the formation of a distinct stacked configuration, likely originating from the unique solvation and/or coordination environment provided by DMF during the synthesis.

**Fig. 3:**
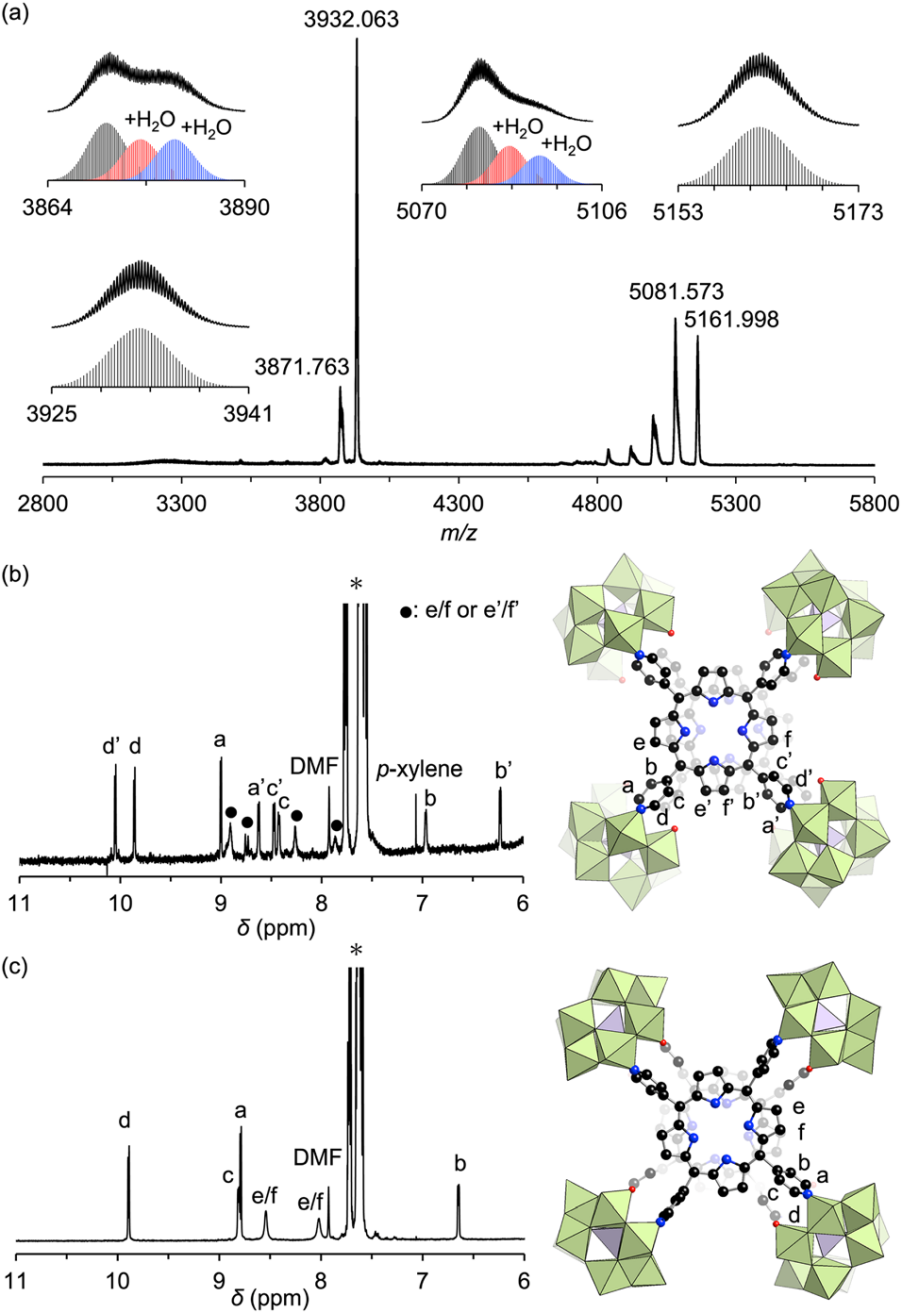
(a) ESI-mass spectrum of hybrid II in acetonitrile. Insets: (top) observed spectra in *m*/*z* ranges of 3864–3890, 3925–3941, 5070–5106, and 5153–5173; (bottom) simulated patterns for [TBA_19_H(SiW_10_O_34_)_4_(H_2_TPyP)_2_(H_2_O)_*n*_]^4+^ (*n* = 0–2), [TBA_20_(SiW_10_O_34_)_4_(H_2_TPyP)_2_]^4+^, [TBA_18_H(SiW_10_O_34_)_4_(H_2_TPyP)_2_(H_2_O)_*n*_]^3+^ (*n* = 0–2), and [TBA_19_(SiW_10_O_34_)_4_(H_2_TPyP)_2_]^3+^. (b and c) ^1^H NMR spectra of the tetraphenylphosphonium salt of hybrid I (b) and hybrid II (c) in acetonitrile-*d*_3_ (asterisk symbols indicate tetraphenylphosphonium ions).

Adding ethyl acetate to the DMF reaction mixture facilitated crystallization, producing brown crystals of hybrid II with a 30% yield. The UV-vis spectrum of this hybrid in acetonitrile displayed a Soret band at 399 nm, consistent with the above reaction mixture, confirming that hybrid II was formed as the major product (Fig. S2†). The electrospray ionization mass (ESI-mass) spectrum of hybrid II in acetonitrile displayed sets of signals at mass-to-charge ratio (*m*/*z*) values of 3871.763, 3932.063, 5081.573, and 5161.998, corresponding to [TBA_19_H(SiW_10_O_34_)_4_(H_2_TPyP)_2_]^4+^ (theoretical *m*/*z*: 3871.787), [TBA_20_(SiW_10_O_34_)_4_(H_2_TPyP)_2_]^4+^ (theoretical *m*/*z*: 3932.106), [TBA_18_H(SiW_10_O_34_)_4_(H_2_TPyP)_2_]^3+^ (theoretical *m*/*z*: 5081.621), and [TBA_19_(SiW_10_O_34_)_4_(H_2_TPyP)_2_]^3+^ (theoretical *m*/*z*: 5162.047), respectively (Fig. 3a). Additional signals attributed to hydrated species [TBA_19_H(SiW_10_O_34_)_4_(H_2_TPyP)_2_(H_2_O)_*n*_]^4+^ (*n* = 1 or 2) and [TBA_18_H(SiW_10_O_34_)_4_(H_2_TPyP)_2_(H_2_O)_*n*_]^3+^ (*n* = 1 or 2) were also observed. These UV-vis and ESI-mass results confirmed the formation of an isomer with the same molecular composition as hybrid I.

Single crystals suitable for X-ray diffraction analysis were obtained by recrystallizing hybrid II as a tetraphenylphosphonium salt (see ESI for details†). X-ray crystallographic analysis revealed structural similarities between hybrids I and II: specifically, both hybrids comprised two stacked porphyrins coordinated to four {SiW_10_} units at the corners (Fig. 2a, b, Tables S2 and S3†). However, hybrid II contained porphyrins aligned without lateral displacement, with an interfacial distance of 3.6 Å—slightly longer than the distance of 3.4 Å observed in hybrid I—and a rotational displacement of 24°. Conversely, hybrid I featured a lateral displacement of 1.1 Å without rotational displacement. Additionally, hybrid I comprised two distinct coordination modes of {SiW_10_} units, whereas hybrid II comprised a single mode. These structural variations underscore the potential of lacunary POMs to modulate the lateral displacement, rotational displacement, and interfacial distance of porphyrins by altering the coordination mode of pyridyl groups to the {SiW_10_} units. Notably, the short interfacial distances, ideal for π–π interactions, were effectively achieved in both hybrids using {SiW_10_} linkers. The proton nuclear magnetic resonance (^1^H NMR) spectrum of the tetraphenylphosphonium salt of hybrid II displayed six peaks in the aromatic region (6.64–9.89 ppm), attributed to its higher molecular symmetry compared to that of hybrid I (Fig. 3b and c). Density functional theory (DFT) calculations revealed that hybrid II is thermodynamically more stable than hybrid I, exhibiting an energy difference of 35.2 kJ mol^−1^. Notably, heating hybrid I in DMF or hybrid II in DMA/DCE (1/1, v/v) at 80 °C for 2 h resulted in no changes to their Soret bands, demonstrating that both hybrids are stable and do not interconvert under these conditions (Fig. S3†). While previously reported methods for synthesizing cofacial porphyrin dimers using organic linkers^6^ or metal-ion linkers^7^ typically produce a single dimer configuration, our strategy employing lacunary POMs provides a versatile approach to modulate key structural parameters while maintaining a consistent molecular framework.

**Fig. 4:**
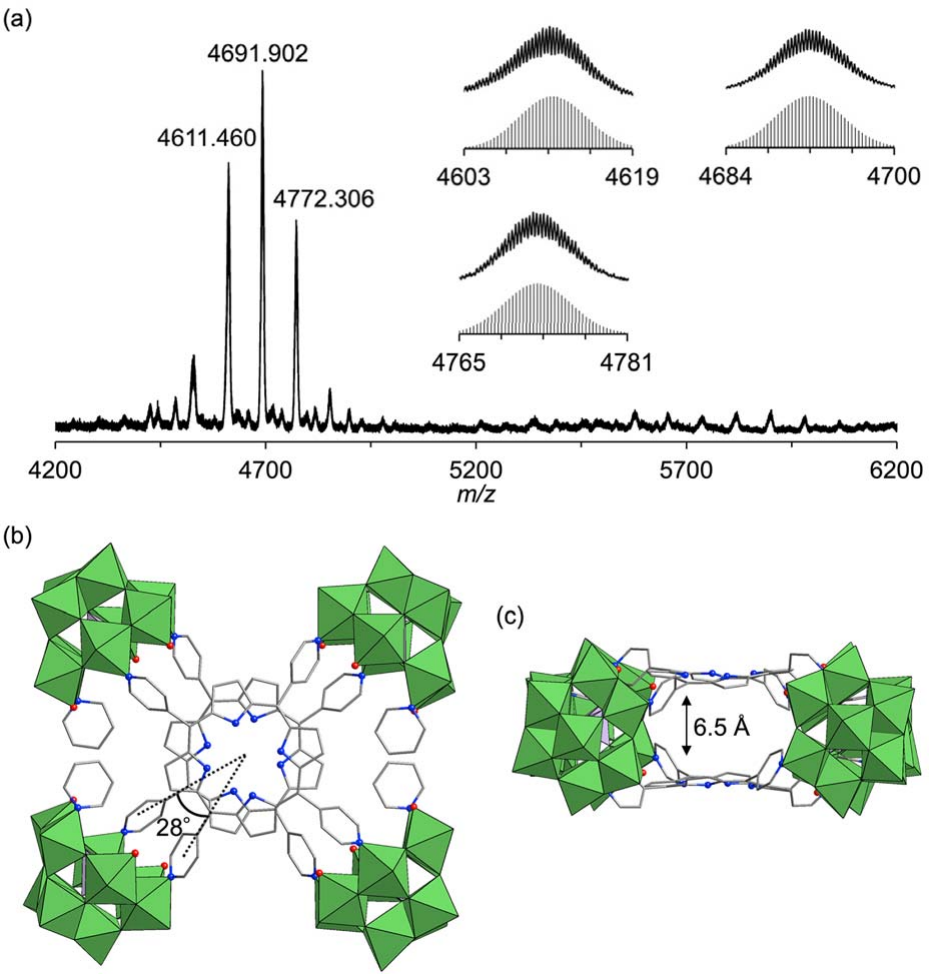
(a) ESI-mass spectrum of hybrid III in a mixture of acetone/pyridine (99/1, v/v). Insets: (top) observed spectra in *m*/*z* ranges of 4603–4619, 4765–5781, and 4684–4700; (bottom) simulated patterns for [TBA_16_H_3_(SiW_9_O_31_)_4_(H_2_TPyP)_2_]^3+^, [TBA_17_H_2_(SiW_9_O_31_)_4_(H_2_TPyP)_2_]^3+^, and [TBA_18_H(SiW_9_O_31_)_4_(H_2_TPyP)_2_]^3+^. (b and c) Crystal structure of the anionic part of hybrid III ((b) top view; (c) side view). Color code: W, green; Si, purple; C, gray; N, blue; O, red. H atoms are omitted for clarity.

Next, to synthesize porphyrin dimers with an extended interfacial distance compared to hybrids I and II, we utilized a trivacant lacunary polyoxotungstate, [SiW_9_O_34_]^10−^, which offers coordination sites for pyridine spaced 6.2–6.5 Å apart^14^—considerably farther than those of [SiW_10_O_36_]^8−^ (5.5–5.8 Å). The reaction of TBA_4_[SiW_9_O_28_(OCH_3_)_6_]^14^ with H_2_TPyP in a 2 : 1 molar ratio in a mixture of DMA/chloroform/pyridine (50/50/1, v/v) at 50 °C for 2 h, followed by the addition of toluene, yielded purple crude solids. Recrystallizing these solids in DCE/*p*-xylene with pyridine (100 equivalents relative to III) afforded purple single crystals of hybrid III (see ESI for details†). The ESI-mass spectrum of hybrid III in an acetone/pyridine mixture (99/1, v/v) exhibited distinct sets of signals at *m*/*z* values of 4611.460, 4691.902, and 4772.306, corresponding to [TBA_16_H_3_(SiW_9_O_31_)_4_(H_2_TPyP)_2_]^3+^ (theoretical *m*/*z*: 4611.522), [TBA_17_H_2_(SiW_9_O_31_)_4_(H_2_TPyP)_2_]^3+^ (theoretical *m*/*z*: 4691.947), and [TBA_18_H(SiW_9_O_31_)_4_(H_2_TPyP)_2_]^3+^ (theoretical *m*/*z*: 4772.373), respectively (Fig. 4a). Single-crystal X-ray structural analysis revealed that hybrid III consists of an anionic structure featuring two stacked porphyrins coordinated to four {SiW_9_} units at the corners. The interfacial distance between the porphyrins was 6.5 Å, substantially longer than those observed in hybrids I (3.4 Å) and II (3.6 Å) (Fig. 4b, c, Tables S2 and S4†). Additionally, hybrid III exhibited a rotational displacement of 28° between the porphyrins.

### Optical properties

**Fig. 5:**
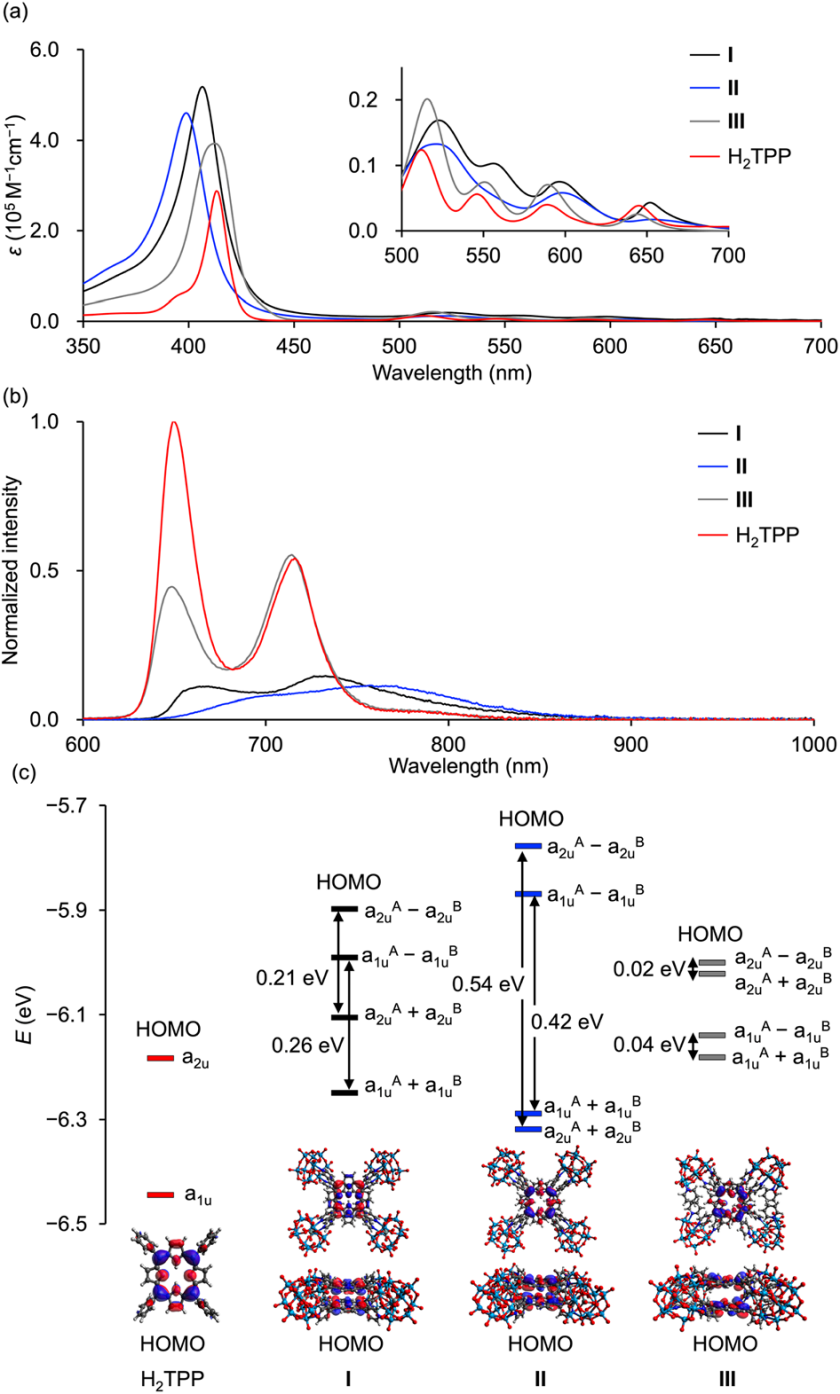
Optical properties of hybrids I, II, and III and monomeric H_2_TPP. (a) UV-vis spectra in acetonitrile (I, 2.5 μM; II, 2.5 μM; III, 2.5 μM; H_2_TPP, 5 μM). Inset: enlarged Q-band region. (b) Fluorescence spectra of I, II, and III and H_2_TPP (excitation at Soret band: I, 406 nm; II, 399 nm; III, 412 nm; H_2_TPP, 413 nm) in acetonitrile. The samples were prepared so that the absorbance of Soret band was 1.0 in acetonitrile. (c) DFT-derived energy diagram from the highest occupied molecular orbital (HOMO) to HOMO−3 of I, II, III, and H_2_TPP and ball-and-stick representation of the HOMO. Energy splitting of molecular orbitals is indicated using double-headed arrows.

The UV-vis spectra of hybrids I, II, and III, along with monomeric H_2_TPP, in acetonitrile displayed Soret bands at 406, 399, 412, and 413 nm, respectively (Fig. 5a). The cofacial arrangement of porphyrin dimers induces exciton interactions between two large transition electric dipoles of porphyrins, causing energy level splitting and a corresponding blue shift in the Soret band, as transitions to the split lower-energy state are forbidden.^5*d*^ The more pronounced blue shift observed for hybrid II compared to hybrid I and H_2_TPP (Table S5†) reflects its stronger exciton interactions, which are attributed to the tighter, non-laterally displaced stacking of porphyrins. In contrast, the weak exciton interactions in hybrid III, resulting from the increased porphyrin separation (6.5 Å), produced a Soret band closely resembling that of monomeric H_2_TPP. The splitting energy (Δ*E*), calculated using Δ*E* = (2|*M*|(1 − 3 cos^2^*φ*))/*x*^3^ (where *M* denotes the transition dipole moments of porphyrins, *x* represents the distance between porphyrin centers, and *φ* denotes the angle between porphyrin centers),^5*d*^ followed the trend II > I > III (Table S6†). This order is consistent with the observed degree of blue shift in the Soret bands. In the Q-bands, exciton interactions are negligible due to small transition electric dipoles. The Q-bands of all hybrids were red-shifted compared to those of H_2_TPP, with hybrid II exhibiting the most pronounced shift. This trend correlates with stabilization of the lowest excited singlet (S_1_) state in cofacial porphyrin dimers, driven by enhanced charge transfer (CT) configurations from the increased orbital overlap between porphyrins.^5*a*,*b*^ The pronounced red shift in the Q-bands of hybrid II reflects the mixing of CT configurations with localized excited states, attributed to the strong orbital overlap between porphyrins. In contrast, the minimal red shift in hybrid III reflects the negligible orbital overlap due to the increased porphyrin separation.

The fluorescence spectrum of hybrid II further substantiated the enhanced contribution of CT configurations. The fluorescence peak of hybrid II (706 nm) appeared significantly red-shifted compared to those of hybrid I (668 nm), hybrid III (648 nm), and H_2_TPP (649 nm), highlighting its greater mixing with CT configurations (Fig. 5b and Table S7,† excitation at Soret band: I, 406 nm; II, 399 nm; III, 412 nm; H_2_TPP, 413 nm in acetonitrile). The structureless and broad fluorescence spectrum of hybrid II also reflects the enhanced contribution of CT configurations. Similarly, the phosphorescence peak of hybrid II demonstrated a greater red shift compared to those of hybrid I, hybrid III, and H_2_TPP, further corroborating the enhanced stabilization of its lowest excited triplet (T_1_) through CT configurations (Fig. S4†).^5*a*^ Hybrid II also demonstrated greater splitting of the a_2u_ and a_1u_ orbitals (0.54 and 0.42 eV, respectively) compared to hybrid I (0.21 and 0.26 eV), reflecting the stronger orbital overlap between its porphyrins and stronger π–π interactions owing to its non-laterally displaced cofacial stacking (Fig. 5c and S5†).^5*a*^ In contrast, hybrid III showed minimal splitting (0.02 eV and 0.04 eV), consistent with the lack of orbital overlap resulting from its increased interfacial distance. Cyclic voltammetry revealed a lower oxidation potential of the porphyrins in hybrid II (0.85 V *vs.* Ag/Ag^+^) compared to those in hybrid I (0.87 V *vs.* Ag/Ag^+^) and H_2_TPyP (1.00 V *vs.* Ag/Ag^+^) (Fig. S6a†). This trend aligns with the order of the HOMO energy levels: hybrid II > hybrid I > monomeric H_2_TPP (Fig. 5c). Furthermore, the reduction potential of the porphyrins in hybrid II (−1.47 V *vs.* Ag/Ag^+^) was more negatively shifted compared to that of hybrid I (−1.35 V *vs.* Ag/Ag^+^) and H_2_TPyP (−1.30 V *vs.* Ag/Ag^+^) (Fig. S6b†). The fluorescence quantum yield (*Φ*_F_) of hybrid II (*Φ*_F_ = 0.019) was slightly lower than that of hybrid I (*Φ*_F_ = 0.023, Table 1), likely attributed to increased thermal deactivation from S_1_ to S_0_, facilitated by the greater contribution of CT configurations.^15^ Furthermore, the quantum yield for 
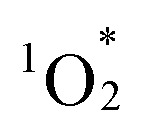
 generation (*Φ*_Δ_) in hybrid II (*Φ*_Δ_ = 0.50) was lower than that in hybrid I (*Φ*_Δ_ = 0.76, Table 1), also attributable to enhanced thermal deactivation. Nonetheless, the *Φ*_Δ_ value of hybrid II remained comparable to that of H_2_TPP, likely owing to the heavy atom effect imparted by the POM units.

**Table 1:** Quantum yields for 
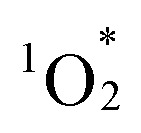
 generation (*Φ*_Δ_) and fluorescence (*Φ*_F_)

Compound	*Φ*_Δ_ a	*Φ*_F_
I	0.76	0.023
II	0.50	0.019
III	0.56	0.049
H_2_TPP	0.50	0.060

a *Φ*_Δ_ was determined using H_2_TPP (*Φ*_Δ_ = 0.50) as a reference.

### Photosensitized aerobic oxidation reactions and stabilities

Photosensitized aerobic oxidation reactions using hybrids I, II, and III were assessed under visible light irradiation (*λ* > 400 nm) using a xenon lamp with a 400 nm cutoff filter as the light source and oxygen (1 atm) as the oxidant. The substrate α-terpinene (1a) was selected owing to its ability to differentiate reactive oxygen species based on the products formed: ascaridole (1b) signifies the involvement of 
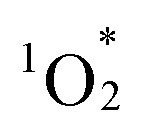
, while *p*-cymene (1c) suggests the participation of other reactive oxygen species, such as the superoxide radical anion (O_2_˙^−^), hydroxyl radical (˙OH), and hydrogen peroxide (H_2_O_2_).^16^ In the presence of a catalytic amount of hybrid II (0.003 μmol, 0.003 mol% with respect to 1a), 1a was efficiently converted to 1b with an 87% yield after just 4.5 min of visible light irradiation, with negligible formation of 1c (Table 2, entry 1). This result confirms that 
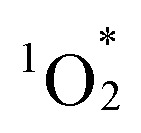
 is the primary reactive oxygen species in this system. Hybrid II exhibited superior activity compared to H_2_TPP, despite both displaying similar 
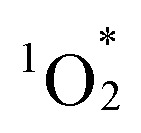
 generation efficiencies (Table 2, entry 2 *vs.* entry 4). When H_2_TPP and TBA_4_H_4_[SiW_10_O_36_] were used together, the observed activity resembled that of H_2_TPP alone, emphasizing the critical role of porphyrin–POM hybridization in boosting the performance of photosensitized reaction (Table 2, entry 4 *vs.* entry 5). Conversely, hybrid III displayed lower performance than H_2_TPP (Table 2, entry 3 *vs.* entry 4). Furthermore, hybrid II exhibited slightly lower performance than I, consistent with its lower 
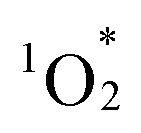
 generation efficiency (Table 2, entry 2 *vs.* entry 6).

**Table 2:** Effect of catalysts on the aerobic oxidation of α-terpinene (1a) under visible light irradiation^a^

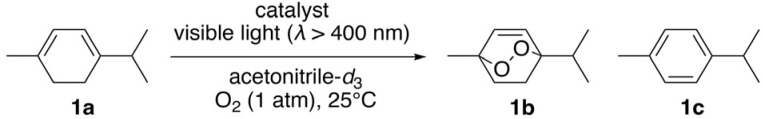				
Entry	Catalyst	Conv. (%)	Yield (%)	
			1b	1c
1^b^	II	>99	87	<1
2	II	61	53	<1
3	III	21	16	<1
4	H_2_TPP	43	35	<1
5	H_2_TPP + TBA_4_H_4_[SiW_10_O_36_]	42	34	<1
6	I	82	72	<1

a Reaction conditions: 1a (0.1 mmol), catalyst (I, II, and III: 0.003 μmol; H_2_TPP: 0.006 μmol; TBA_4_H_4_[SiW_10_O_36_]: 0.012 μmol), acetonitrile-*d*_3_ (0.7 mL), visible light (*λ* > 400 nm), 25 °C, O_2_ (1 atm), 3 min. Product yields and conversion were determined by ^1^H NMR using anisole as an internal standard.

b 4.5 min.

**Fig. 6:**
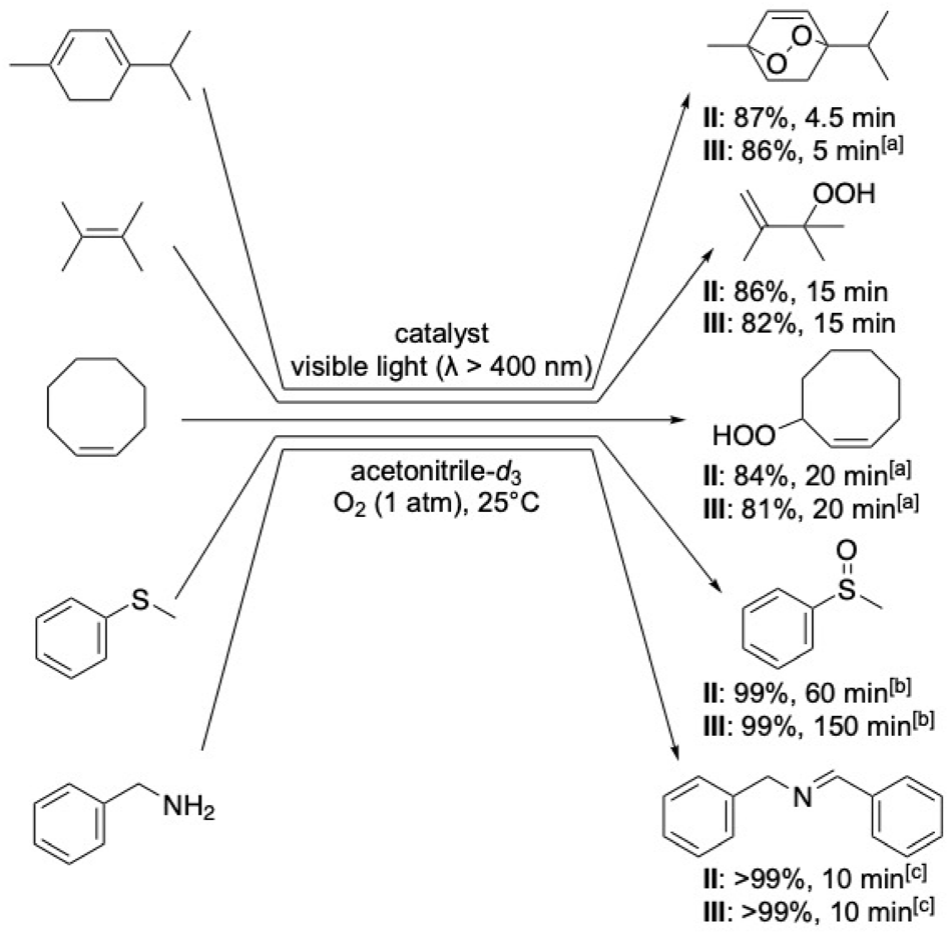
Aerobic oxidation of various substrates using hybrids II and III under visible light irradiation. Reaction conditions: substrate (0.1 mmol), catalyst (II and III: 0.003 μmol), acetonitrile-*d*_3_ (0.7 mL), visible light (*λ* > 400 nm), 25 °C, O_2_ (1 atm). Product yields and conversion were determined by ^1^H NMR using anisole as an internal standard. ^*a*^Catalyst (0.03 μmol). ^*b*^Acetonitrile/water (2 mL, 95/5, v/v). GC yields using dodecane as an internal standard. ^*c*^Acetonitrile (2 mL). GC yields using dodecane as an internal standard.

Next, we examined the substrate scope for the aerobic oxidation reactions using hybrids II and III under visible light irradiation. By using hybrids II and III, 2,3-dimethyl-2-butene, cyclooctene, thioanisole, and benzylamine were efficiently converted to 2,3-dimethylbut-3-en-2-ylhydroperoxide, cyclooct-2-ene-hydroperoxide, methylphenylsulfoxide, and *N*-benzylidenebenzylamine, respectively (Fig. 6).

**Fig. 7:**
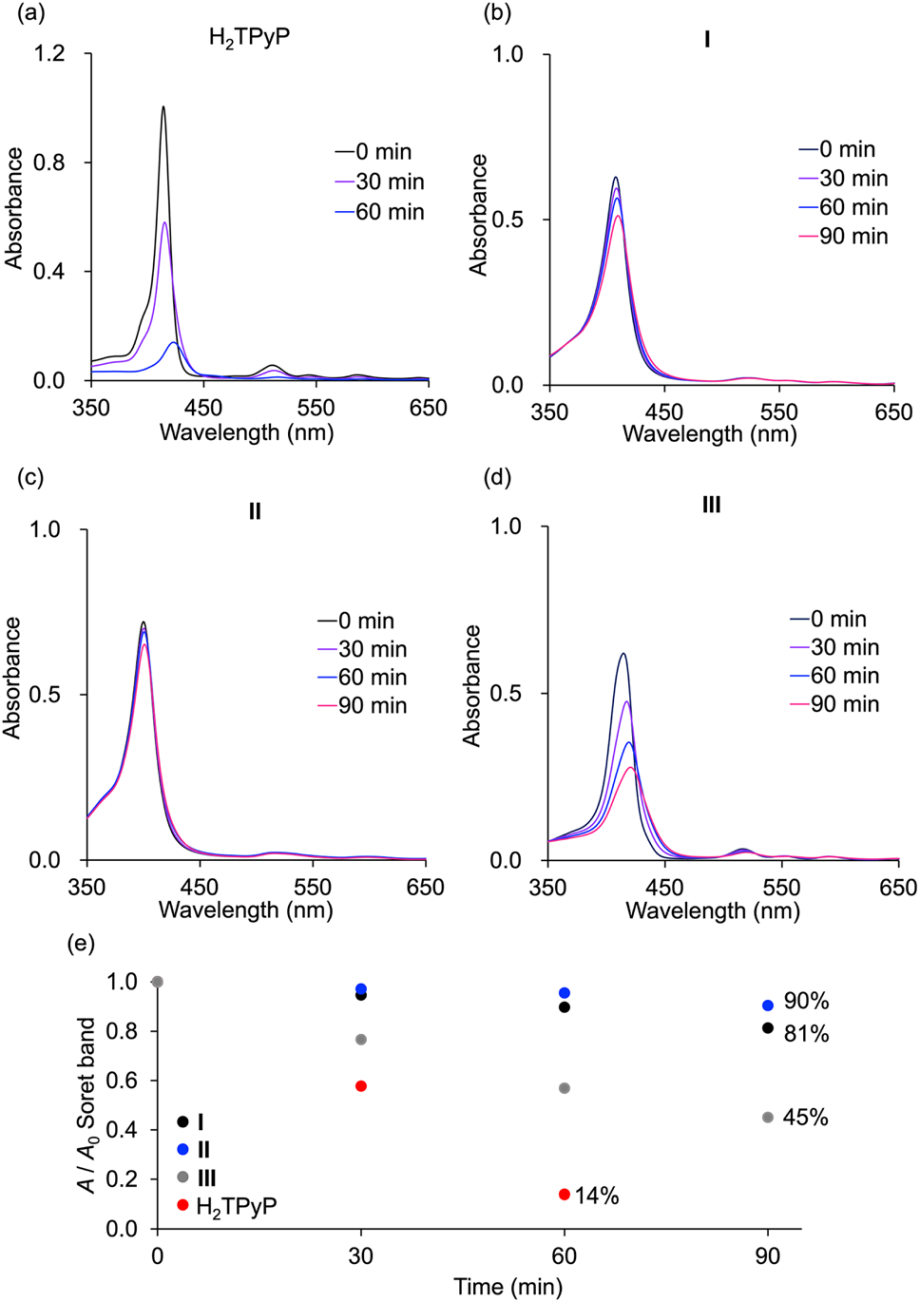
Time-dependent absorption spectra of (a) H_2_TPyP and (b) hybrid I, (c) hybrid II, and (d) hybrid III in acetonitrile/dichloromethane (1/1, *v*/*v*) upon photoirradiation (*λ* > 350 nm) in air. (e) Changes in the *A*/*A*_0_ values of the Soret band of hybrids I, II, and III and H_2_TPyP (*A*: Soret band intensity; *A*_0_: initial Soret band intensity).

The durability of hybrids I, II, and III, along with monomeric H_2_TPyP, was evaluated under photoirradiation in air in a mixture of acetonitrile/dichloromethane (1/1, v/v). Monomeric H_2_TPyP experienced noticeable degradation, with its Soret band absorbance ratio (*A*/*A*_0_; where *A* represents the Soret band absorbance, and *A*_0_ denotes the initial absorbance) dropping to 14% after 60 min (Fig. 7). In contrast, hybrids I and II demonstrated remarkable stability, retaining over 90% of their initial absorbance under identical conditions. Meanwhile, hybrid III exhibited moderate stability. Notably, after 90 min of photoirradiation, hybrid II exhibited superior durability compared to hybrid I (*A*/*A*_0_ = 90% for hybrid II and 81% for hybrid I). It is important to highlight that the HOMO levels of hybrids I and II were higher than those of hybrid III and H_2_TPP (Fig. 5c), suggesting that hybrids I and II would be more prone to oxidation. However, contrary to this expectation, they demonstrated exceptionally high stability, which can be attributed to their structural characteristics. The primary degradation pathway of porphyrins involves 
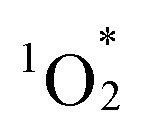
 addition to the C=C double bonds at the meso-position, forming peroxide intermediates and subsequently cleaving C–C bonds.^17^ This process converts sp^2^ carbons to sp^3^, distorting the porphyrin framework. The enhanced stability of hybrids I and II is attributed to the synergistic effects of POM coordination and the strong π–π interactions between stacked porphyrins, which stabilize the structure and suppress 
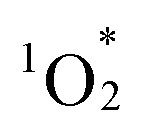
 attacks. The stronger π–π interactions in hybrid II further boost its resistance to degradation, explaining for its superior durability compared to hybrid I.

DFT calculations corroborated the experimental findings by providing estimates of the activation energy for 
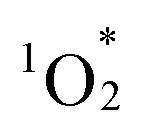
 addition. According to the results, hybrid II exhibited a slightly higher activation energy than hybrid I, reflecting the inhibitory effects of strong π–π interactions on 
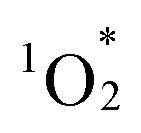
 attacks in hybrid II (Fig. S7†). In contrast, hybrid III demonstrated a lower activation energy than hybrids I and II, consistent with its lack of π–π interactions, making its porphyrin rings more vulnerable to distortion and subsequent degradation. However, the activation energy for hybrid III remained higher than that for H_2_TPyP, likely owing to the structural rigidity imparted by POM coordination. These findings underscore the dual contributions of POM coordination and π–π interactions in enhancing the resistance of porphyrin dimers to 
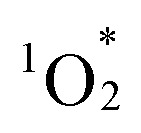
-induced degradation. Because of the high durability, resulting from the dual contributions of POM coordination and π–π interactions, the structure of hybrid II was maintained even after the photo-oxidation of 1a under visible light irradiation: The ESI-mass spectrum of the retrieved hybrid II after the photo-oxidation of 1a exhibited distinct sets of signals at *m*/*z* 3932.080, and 5161.927 assignable to [TBA_20_(SiW_10_O_34_)_4_(H_2_TPyP)_2_]^4+^ (theoretical *m*/*z*: 3932.106), and [TBA_19_(SiW_10_O_34_)_4_(H_2_TPyP)_2_]^3+^ (theoretical *m*/*z*: 5162.047), respectively (Fig. S8†).

## Conclusions

This study demonstrates the successful engineering of cofacial porphyrin dimers using multivacant lacunary POMs as versatile linkers, allowing precise control over key structural parameters, including lateral displacement, rotational displacement, and interfacial distance between individual porphyrins. Hybrid II, synthesized under distinct reaction conditions but at the same molecular composition as previously reported hybrid I (*i.e.*, four divacant lacunary [SiW_10_O_36_]^8−^ and two H_2_TPyP),^13^ exhibited a distinctive porphyrin dimer configuration with no lateral displacement, a rotational displacement of 24°, and an interfacial distance of 3.6 Å. Conversely, hybrid I featured a lateral displacement of 1.1 Å, a further shorter interfacial distance of 3.4 Å, and no rotational displacement. Meanwhile, substituting [SiW_10_O_36_]^8−^ with trivacant [SiW_9_O_34_]^10−^ enabled hybrid III to achieve a significantly extended interfacial distance of 6.5 Å and an increased rotational displacement of 28°, demonstrating the tunability of porphyrin stacking configurations through POM design. These hybrids demonstrated efficient visible-light-responsive photosensitized reactions to generate singlet oxygen 
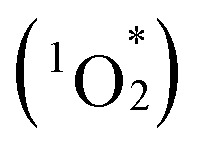
 from ground-state triplet oxygen (^3^O_2_), leading to the photooxidation of various organic substrates. The stronger porphyrin–porphyrin interactions in hybrid II led to pronounced changes in its optical properties, including UV-vis absorption, fluorescence, and phosphorescence, as well as greater resistance to 
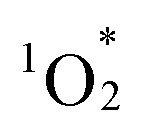
-induced degradation. These findings underscore the originality and potential of lacunary POMs as precise tools for spatially engineering porphyrin dimers, establishing a versatile platform for developing advanced materials with tailored photophysical and catalytic functionalities. We believe that this study establishes a robust platform for engineering porphyrin-based architectures with potential applications in the fields of photocatalysis, energy conversion, and materials science.

## Data availability

The data supporting this manuscript is available in the ESI of and available on request.† Crystallographic data have been deposited at the CCDC (deposition numbers 2417813 and 2417814) and can be obtained free of charge from Cambridge Crystallographic Data Centre *via*http://www.ccdc.cam.ac.uk/data_request/cif.

## Author contributions

M. Y. performed main parts of experiments, including synthesis, characterization, DFT calculations, and photochemical reactions. M. Y. and K. Su. performed DFT calculations. M. Y. and K. Yo. performed crystallographic analysis. K. Sh. and C. L. performed synthesis. K. I. and K. M. performed photophysical measurements. K. Su., K. Ya., K. I. design the project and experiments.

## Conflicts of interest

There are no conflicts to declare.

## Supplementary Material

SC-OLF-D5SC00814J-s001

SC-OLF-D5SC00814J-s002
