# Supplementary material for: Engineering cofacial porphyrin dimers using lacunary polyoxotungstates
Source: Chem Sci. 2025 Apr 22;16(21):9178–85. doi: 10.1039/d5sc00814j (PMC12036599; doi:10.1039/d5sc00814j)
Supplement: SC-016-D5SC00814J-s001 [file SC-016-D5SC00814J-s001.pdf]

Supplementary Information (SI) for Chemical Science.
This journal is © The Royal Society of Chemistry 2025

Electronic Supplementary Information (ESI) for

Engineering cofacial porphyrin dimers using lacunary polyoxotungstates

Masahiro Yamaguchi,[a] Kentaro Yonesato,[a] Kaito Shioya,[a] Chifeng Li,[a] Kei Murata,[b,c] Kazuyuki Ishii,*[,b] Kazuya Yamaguchi*[,a] and Kosuke Suzuki*[,a]

[a] Department of Applied Chemistry, School of Engineering, The University of Tokyo, 7-3-1 Hongo, Bunkyo-ku, Tokyo 113-8656, Japan.

[b] Institute of Industrial Science, The University of Tokyo, 4-6-1 Komaba, Meguro-ku, Tokyo, 153-8505, Japan.

[c] RIKEN Center for Sustainable Resource Science, 2-1 Hirosawa, Wako-shi, Saitama, 351-0198, Japan.

Contents

Page

1. Experimental section S2–S5
2. Tables S1–S7 .... S6–S8
3. Figs. S1–S9 .... S9–S14
4. References S14

Experimental section

Instruments

ESI-mass spectra were recorded on a Shimadzu LCMS 9050 instrument and a Waters Xevo G2-XS QTof instrument. IR spectra were measured on a JASCO FT/IR-4100 spectrometer using KBr disks. UV–vis spectra were measured on a Jasco V-770 spectrometer with a quartz cell of 1 cm path length. Fluorescence spectra and quantum yields were measured on an Otsuka Electronics QE-2000 spectrometer with excitation at the Soret bands (**I**, 406 nm; **II**, 399 nm; **III**, 412 nm; H_2TPP, 413 nm). Gas chromatography (GC) analyses were performed on a Shimadzu GC-2014 instrument with a flame ionization detector equipped with a TC-1 capillary column. NMR spectra were recorded on a JEOL JNM ECA-500 spectrometer (^1H, 500.16 MHz; ^{13}C, 125.77 MHz) using 5 mm tubes. Chemical shifts (δ) are reported in ppm downfield from tetramethylsilane. Cyclic voltammetry measurements were performed with a BioLogic VSP-300 at the scan rate of 50 mV s^{-1}. A standard three-electrode arrangement was employed with a BAS glassy carbon disk electrode as the working electrode, a platinum wire as the counter electrode, and a silver wire electrode as the pseudoreference electrode. The potentials were measured using Ag/$AgNO_3$ reference electrode (10 mM $AgNO_3$, 100 mM $TBAClO_4$ in acetonitrile). Thermogravimetric and differential thermal analyses were performed using a Rigaku Thermo plus EVO2 TG-DTA8122 instrument. Elemental analyses for C, H and N were performed using a MICRO CORDER JM10 at Research Infrastructure Management Center Core Facility Center of Institute of Science Tokyo. Inductively coupled plasma atomic emission spectroscopy analyses for W and Si were performed with a Shimadzu ICPS-8100 instrument.

Materials

Tetraphenylphosphonium bromide (TCI), *N*,*N*-dimethylacetamide (DMA, TCI), *N*,*N*-dimethylformamide (DMF, Kanto Chemical), chloroform (Kanto Chemical), pyridine (Kanto Chemical), nitromethane (TCI), ethyl acetate (Kanto Chemical), acetone (Kanto Chemical), 1,2-dichloroethane (DCE, Kanto Chemical), acetonitrile (Kanto Chemical), toluene (Kanto Chemical), *p*-xylene (TCI), dichloromethane (Kanto Chemical), 5,10,15,20-tetra(4-pyridyl)porphyrin (H_2TPyP, Sigma-Aldrich), 5,10,15,20-tetraphenylporphyrin (H_2TPP, Sigma-Aldrich), tetrabutylammonium perchlorate ($TBAClO_4$, Nacalai Tesque), acetonitrile-d_3 (TCI), α-terpinene (Wako), dodecane (TCI), anisole (TCI), 2,3-dimethyl-2-butene (TCI), cyclooctene (TCI), benzylamine (TCI), and thioanisole (TCI) were used as received. The syntheses of $TBA_4H_4[SiW_{10}O_{36}]$, $TBA_{16}[(SiW_{10}O_{34})_4(H_2TPyP)_2]$ (hybrid **I**), and $TBA_4[SiW_9O_{28}(OCH_3)_6]$ were performed according to the reported procedures and confirmed by ESI-mass spectrometry and/or UV–vis spectroscopy.[S1,S2]

X-ray crystallography

Single-crystal X-ray diffraction measurements were performed at BL02B1 beamline at the SPring-8 facility of the Japan Synchrotron Radiation Research Institute (λ = 0.4124 or 0.4132 Å, monochromatized

by a Si(311) double-crystal monochromator) with a PILATUS3 X CdTe 1M detector at −173°C. The data collection and process were conducted using RAPID-AUTO and CrysAlisPro software.[S3] In the reduction of data, Lorentz and polarization corrections were made. The structural analyses were performed using Olex2 and WinGX.[S4] Structures were solved using SHELXT-2018/2 (intrinsic phase methods)[S5] and refined by SHELXL-2018/3.[S6] All non-hydrogen atoms (Si, W, O, C and N) were refined anisotropically. Highly disordered cations and solvent molecules were omitted by using SQUEEZE program.[S7] CCDC-2417813 and 2417814 contain the supplementary crystallographic data for hybrids **II** and **III**, respectively. These data can be obtained free of charge from The Cambridge Crystallographic Data Centre via www.ccdc.cam.ac.uk/data_request/cif.

Bond valence sum (BVS) calculations

The BVS values were calculated by the expression for the variation of the length r_{ij} of a bond between two atoms *i* and *j* in observed crystal with valence *Vi* using equation:

$$V_i = \sum_j \exp\left(\frac{r'_0 - r_{ij}}{B}\right)$$

where *B* is a constant equal to 0.37 Å, r'_0 is bond valence parameter for a given atom pair.[S8]

Phosphorescence measurements and determination of $^1O_2$* quantum yield

Phosphorescence measurements were performed using a monochromator (JASCO CT-25CP) and a photomultiplier (Hamamatsu Photonics R5509-43), which was cooled at −80°C by a cold nitrogen gas flow system (Hamamatsu Photonics R6544-20). The photon signals amplified by a fast preamplifier (Stanford Research SR445) were measured by the single photon counting method using a photon counter (Stanford Research SR400). For these measurements, samples were excited by a Nd:YAG laser (Spectra Physics INDI 40; 532 nm; 7 ns fwhm). For the $^1O_2$* phosphorescence measurements, the samples were prepared so that the absorbance of 532 nm was 0.1 in acetonitrile. $^1O_2$* quantum yields (Φ_Δ) were determined by using H_2TPP ($\Phi_\Delta = 0.50$) as a reference.[S9] The phosphorescence measurements of hybrids **I**, **II**, **III**, and monomeric H_2TPP were performed in a mixture of acetonitrile and toluene (1/1, *v*/*v*) at −196°C under air.

DFT calculations

DFT calculations were performed using the Gaussian 16, Rev. B.01. The geometries used in the calculation were based on the crystal structures determined in this study. The structure of hybrids **I**, **II**, and **III** were optimized at the CAM-B3LYP functional with 6-31G* (for C, H, N, O and Si) and LanL2DZ (for W) by using the conductor-like polarizable continuum model with the parameters of the integral equation formalism model for acetonitrile. To account for intermolecular interactions of stacked porphyrins, Grimme's dispersion correction D3 was employed.[S10] The transition states of $^1O_2$* addition to hybrids **I**, **II**, **III** and monomeric H_2TPyP were confirmed by the intrinsic reaction coordinate (IRC)

method. The transition states were also confirmed by imaginary vibrational frequencies.

Synthesis of hybrid II

H_2TPyP (12.4 mg, 20 μmol) was dissolved in DMF (3 mL) and stirred at 50°C for 30 min. $TBA_4H_4[SiW_{10}O_{36}]$ (137 mg, 40 μmol) was added and reacted at 80°C for 6 hours. The mixture was then filtered and crystallized by addition of ethyl acetate to give brown crystals (45 mg, 31%). Single crystals suitable for X-ray crystallography were successfully obtained by recrystallisation of hybrid **II** from a mixture of DMF, DCE, and ethyl acetate in the presence of tetraphenylphosphonium bromide (24 equivalents with respect to hybrid **II**) at 25°C in 7 days. Positive-ion MS (ESI, acetonitrile): *m*/*z* 3871.763 (calcd. 3871.787 for $[TBA_{19}H(SiW_{10}O_{34})_4(H_2TPyP)_2]^{4+}$), 3932.063 (calcd. 3932.106 for $[TBA_{20}(SiW_{10}O_{34})_4(H_2TPyP)_2]^{4+}$), 5081.573 (calcd. 5081.621 for $[TBA_{18}H(SiW_{10}O_{34})_4(H_2TPyP)_2]^{3+}$), 5161.998 (calcd. 5162.047 for $[TBA_{19}(SiW_{10}O_{34})_4(H_2TPyP)_2]^{3+}$. IR (KBr pellet, cm^{-1}): 3440, 3078, 2961, 2933, 2873, 1617, 1559, 1484, 1402, 1382, 1214, 1154, 1107, 1066, 1032, 993, 954, 880, 849, 783, 762, 719, 684, 660, 603, 566, 539, 405, 360. Elemental analysis calcd (%) for $TBA_{15}H(SiW_{10}O_{34})_4(H_2TPyP)_2$ ·3DMF: C 26.81; H, 4.20; N, 3.23; Si, 0.76; W, 49.90. Found: C 26.53, H, 4.06; N, 3.01; Si, 0.73; W, 49.38. UV-vis (acetonitrile solution): λ (ε) 399 nm (4.5×10^5 $M^{-1}cm^{-1}$), 521 nm (1.2×10^4 $M^{-1}cm^{-1}$), 598 nm (5.4×10^3 $M^{-1}cm^{-1}$), 653 nm (1.6×10^3 $M^{-1}cm^{-1}$).

Synthesis of hybrid III

H_2TPyP (12.4 mg, 20 μmol) was dissolved in a mixture of DMA/$CHCl_3$/pyridine (50/50/1, *v*/*v*) (3 mL) and stirred at 50°C for 30 min. $TBA_4[SiW_9O_{28}(OCH_3)_6]$ (131 mg, 40 μmol) was added and reacted at 50°C for 2 hours. The mixture was then filtered and added with toluene to give purple crude solids (100 mg, 72%). Purple crystals of **III** were obtained by recrystallisation of the purple crude solids (100 mg) from a mixture of DCE and *p*-xylene in the presence of pyridine (100 equivalents with respect to **III**) at 25°C in 21 days (30 mg, 30%). Single crystals suitable for X-ray crystallography were successfully obtained by reaction of $TBA_4[SiW_9O_{28}(OCH_3)_6]$ (131 mg, 40 μmol) and H_2TPyP (12.4 mg, 20 μmol) in a mixture of DCE/pyridine (100/3, *v*/*v*) at 50°C for 2 h and adding toluene to the reaction solution. Positive-ion MS (ESI, acetone/pyridine): *m*/*z* 4611.460 (calcd. 4611.522 for $[TBA_{16}H_3(SiW_9O_{31})_4(H_2TPyP)_2]^{3+}$), 4691.902 (calcd. 4691.947 for $[TBA_{17}H_2(SiW_9O_{31})_4(H_2TPyP)_2]^{3+}$), 4772.373 (calcd. 4772.373 for $[TBA_{18}H(SiW_9O_{31})_4(H_2TPyP)_2]^{3+}$). IR (KBr pellet, cm^{-1}): 3435, 2961, 2936, 2873, 1632, 1616, 1562, 1485, 1402, 1381, 1218, 1190, 1154, 1107, 1067, 1027, 990, 947, 888, 804, 741, 684, 641, 560, 520, 453, 374. Elemental analysis calcd (%) $TBA_{15}H(SiW_9O_{31})_4(H_2TPyP)_2$· $(H_2O)_{29}$·*p*-xylene: C 28.76; H, 4.72; N, 3.37; Si, 0.77; W, 45.53. Found: C 28.64, H, 4.68; N, 3.11; Si, 0.70; W, 45.18. UV-vis (acetonitrile solution): λ (ε) 412 nm (3.9×10^5 $M^{-1}cm^{-1}$), 516 nm (2.1×10^4 $M^{-1}cm^{-1}$), 551 nm (7.1×10^3 $M^{-1}cm^{-1}$), 589 nm (7.3×10^3 $M^{-1}cm^{-1}$), 644 nm (2.5×10^3 $M^{-1}cm^{-1}$).

Procedure for the photo-oxidation of α-terpinene, 2,3-dimethyl-2-butene, and cyclooctene

Into a Pyrex Schlenk flask (volume: ~10 mL), α-terpinene or 2,3-dimethyl-2-butene or cyclooctene (0.1 mmol), hybrid **II** (0.003 μmol, 0.003 mol% with respect to α-terpinene/2,3-dimethyl-2-butene or 0.03 μmol, 0.03 mol% with respect to cyclooctene), anisole (0.1 mmol, internal standard), acetonitrile-d_3 (0.7 mL), and a Teflon-coated magnetic stir bar were successively placed under O_2 atmosphere (1 atm). The reaction was initiated by irradiation with visible light (λ > 400 nm) using a xenon lamp (Excelitas Technologies, PE300BFA) equipped with a 400 nm cutoff filter at 25°C. The detailed reaction conditions are described in Table 2 and Fig. 6. The products were confirmed by ^{1}H NMR and the yields and conversion were determined using anisole as an internal standard. After the reaction, hybrid **II** was retrieved by adding an excess amount of toluene to the reaction solution, and the ESI-mass spectrum of the retrieved **II** was measured (Fig. S8): **II** (0.3 μmol, 0.3 mol%), **1a** (0.1 mmol), acetonitrile (0.7 mL), O_2 atmosphere (1 atm), visible light (λ > 400 nm) irradiation at 25°C for 5 min.

Procedure for the photo-oxidation of thioanisole and benzylamine

Into a Pyrex Schlenk flask (volume: ~20 mL), thioanisole or benzylamine (0.1 mmol), hybrid **II** (0.003 μmol, 0.003 mol% with respect to thioanisole/benzylamine), dodecane (0.1 mmol, internal standard), acetonitrile/water (2 mL, 95/5, *v*/*v*) or acetonitrile (2 mL), and a Teflon-coated magnetic stir bar were successively placed under O_2 atmosphere (1 atm). The reaction was initiated at 25°C by irradiation with visible light (λ > 400 nm) from a xenon lamp (Excelitas Technologies, PE300BFA) equipped with a 400 nm cutoff filter. The detailed reaction conditions are described in Fig. 6. The GC yields reported were determined using dodecane as an internal standard.

Table S1. Wavelength of the Soret band of the reaction solution of $TBA_4H_4[SiW_{10}O_{36}]$ and H_2TPyP in various organic solvent (the solution was diluted by acetonitrile for UV-vis measurements).

Reaction solvent	Soret band (nm)
DMF	399
DMA	405
Acetonitrile	406
Nitromethane	406
DMA/DCE	406

Table S2. X-ray crystallographic parameters of hybrids **II** and **III**.

	**II**	**III**
Formula	$C_{320}H_{248}N_{16}O_{136}P_{10}Si_4W_{40}$	$C_{100}H_{68}N_{20}O_{124}Si_4W_{36}$
Fw (g mol^{-1})	14269.39	10264.70
Crystal system	Orthorhombic	Triclinic
Space group	*Pbcn* (No. 60)	*P-1* (No. 2)
a (Å)	32.9135(3)	28.75606(13)
b (Å)	34.9741(2)	31.5544(2)
c (Å)	51.0599(4)	34.30222(19)
α (deg)	90	66.2043(6)
β (deg)	90	85.0443(4)
γ (deg)	90	69.9358(5)
V (Å^3)	58776.1(8)	26701.7(3)
Z	4	2
R_1 [I>2σ(I)]	0.0824	0.0365
wR_2	0.2181	0.1054
GOF	1.064	1.079
ρ_{calc} (g cm^{-3})	1.613	1.277
Temp (K)	100(2)	100(2)

$R_1 = \Sigma||F_o|-|F_c||/\Sigma|F_o|$, $wR_2 = \{\Sigma[w(F_o^2 - F_c^2)]/\Sigma[w(F_o^2)^2]\}^{1/2}$.

Table S3. BVS values for silicon and tungsten atoms of hyrbrid **II**.

Si1A	3.93	Si1B	3.93	Si1C	3.95	Si1D	3.91
W11A	6.17	W11B	6.17	W21C	6.22	W31D	6.62
W12A	6.28	W12B	6.28	W22C	5.97	W32D	5.89
W13A	6.19	W13B	6.19	W23C	6.40	W33D	6.24
W14A	6.08	W14B	6.08	W24C	6.55	W34D	6.44
W15A	6.20	W15B	6.20	W25C	6.18	W35D	6.02
W16A	6.22	W16B	6.22	W26C	6.19	W36D	6.20
W17A	6.41	W17B	6.40	W27C	5.62	W37D	6.09
W18A	6.28	W18B	6.27	W28C	6.18	W38D	5.86
W19A	6.04	W19B	6.04	W29C	6.43	W39D	6.06
W20A	6.18	W20B	6.18	W30C	5.99	W40D	6.35

Table S4. BVS values for silicon and tungsten atoms of hyrbrid **III**.

Si1	3.92	Si2	3.89	Si3	3.90	Si4	3.90
W11	6.14	W21	6.17	W31	6.35	W41	6.15
W12	6.18	W22	6.14	W32	6.10	W42	6.15
W13	6.40	W23	6.23	W33	6.28	W43	6.26
W14	6.10	W24	6.03	W34	6.22	W44	6.16
W15	6.19	W25	6.28	W35	6.17	W45	6.13
W16	6.06	W26	6.28	W36	6.11	W46	6.21
W17	6.24	W27	6.13	W37	6.19	W47	6.20
W18	6.15	W28	6.22	W38	6.24	W48	6.04
W19	6.11	W29	6.18	W39	5.99	W49	6.07

Table S5. Wavelengths of Soret and Q bands in hybrids **I**, **II**, **III** and H_2TPP in acetonitrile.

Compound	Soret band (nm)	Q bands (nm)			
**I**	406	523	556	596	652
**II**	399	521	–	598	653
**III**	412	516	551	589	644
H_2TPP	413	512	546	588	645

Table S6. The relationship between wavelength of Soret band and the splitting energy ΔE.

Compound	Soret band (nm)	x (Å)	φ (°)	ΔE (eV)
**I**	406	3.6	72	$0.015M$
**II**	399	3.7	88	$0.020M$
**III**	412	6.5	88	$0.004M$
H_2TPP	413	–	–	–

$\Delta E = (2|M|(1-3\cos^2\varphi))/x^3$ (where M denotes the transition dipole moments of porphyrins, x represents the distance between porphyrin centers, and φ denotes the angle between porphyrin centers)

Table S7. Wavelengths of fluorescence peaks in hybrids **I**, **II**, **III** and H_2TPP in acetonitrile.

Compound	Fluorescence peaks (nm)	
**I**	668	730
**II**	706	765
**III**	648	714
H_2TPP	649	716

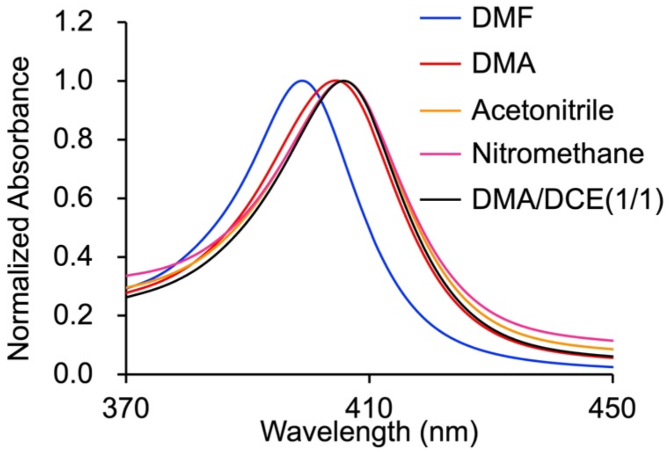

Fig. S1 The UV–vis spectra of the reaction solution of $TBA_4H_4[SiW_{10}O_{36}]$ and H_2TPyP in various organic solvents (DMF, DMA, acetonitrile, nitromethane, DMA/DCE). The spectra were measured by diluting the reaction solutions with acetonitrile (normalized at the peak top of Soret band).

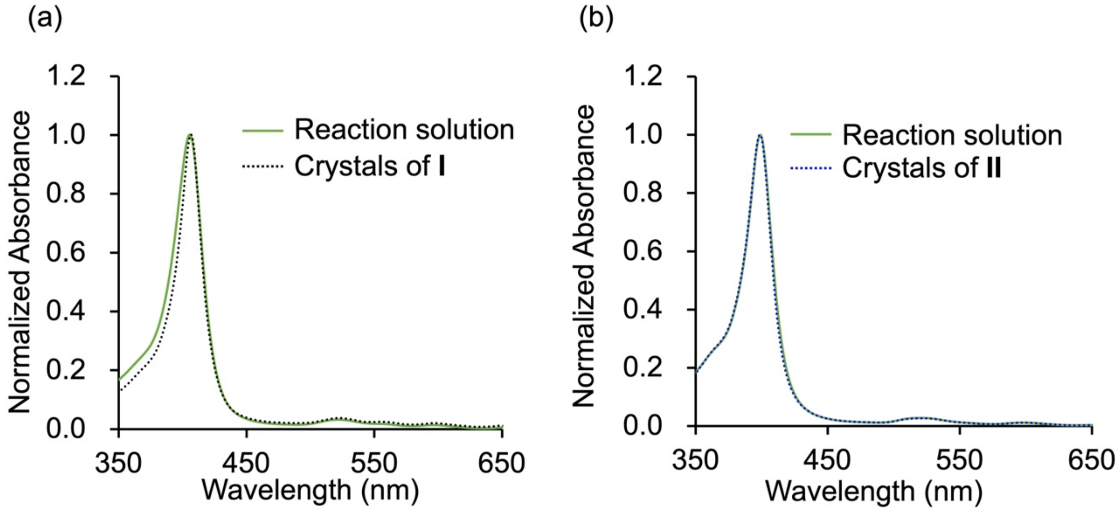

Fig. S2 The comparison of the UV–vis spectra between (a) the reaction solution of $TBA_4H_4[SiW_{10}O_{36}]$ and H_2TPyP in DMA/DCE and crystals of hybrid **I** (1.9 μM), and (b) the reaction solution of $TBA_4H_4[SiW_{10}O_{36}]$ and H_2TPyP in DMF and crystals of hybrid **II** (1.9 μM). The spectra were measured by diluting the reaction solutions with acetonitrile (normalized at the peak top of Soret band).

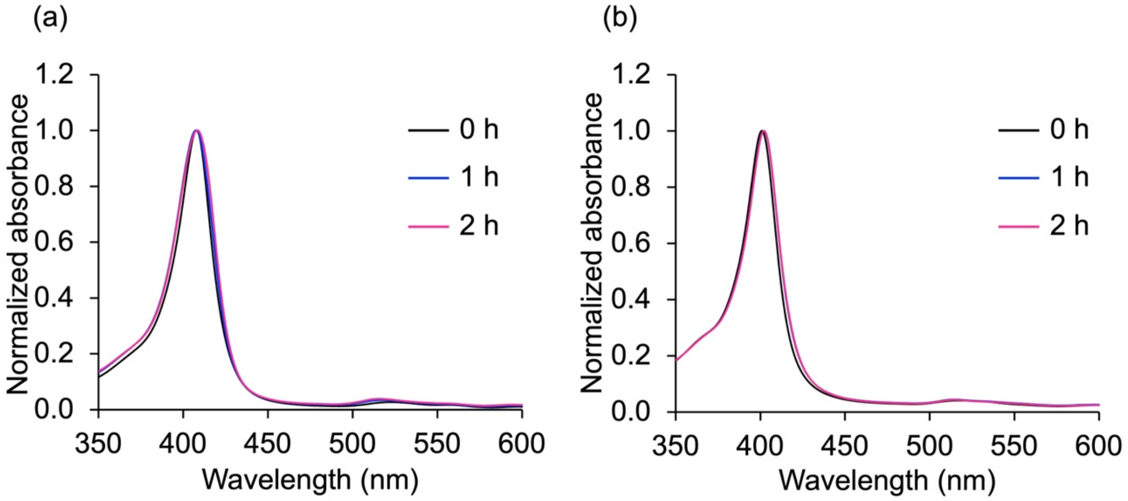

Fig. S3 Time-dependent the UV–vis spectra of (a) hybrid **I** in *N*,*N*-dimethylformamide and (b) hybrid **II** in *N*,*N*-dimethylacetamide/1,2-dichloroethane (1/1, *v*/*v*) heated at 80°C. The spectra were measured by diluting the solutions with acetonitrile (normalized at the peak top of Soret band).

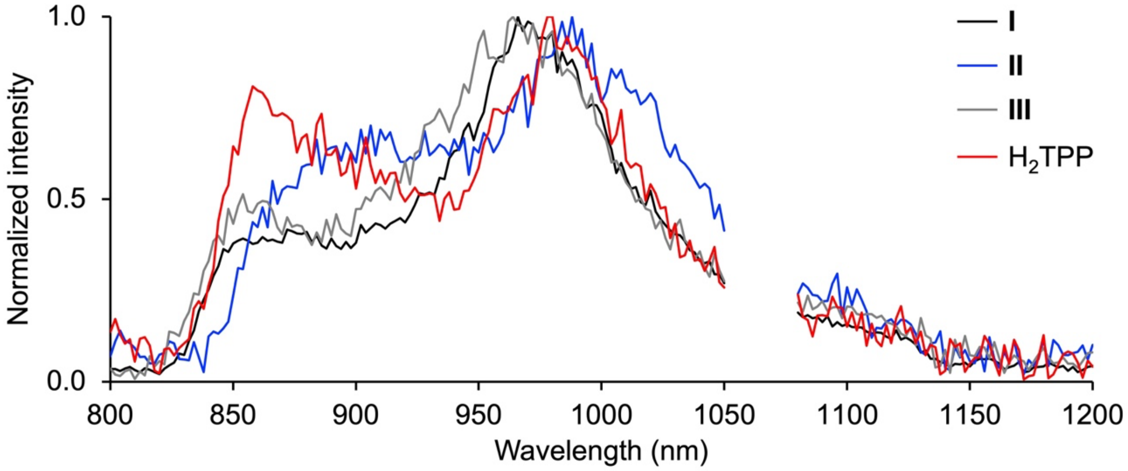

Fig. S4 Phosphorescence spectra of hybrids **I**, **II**, **III**, and monomeric H_2TPP in acetonitrile/toluene (1/1, *v*/*v*) at 77 K upon excitation at 532 nm (**I**, 5.0 μM; **II**, 4.0 μM; **III**, 4.0 μM; H_2TPP, 8.0 μM).

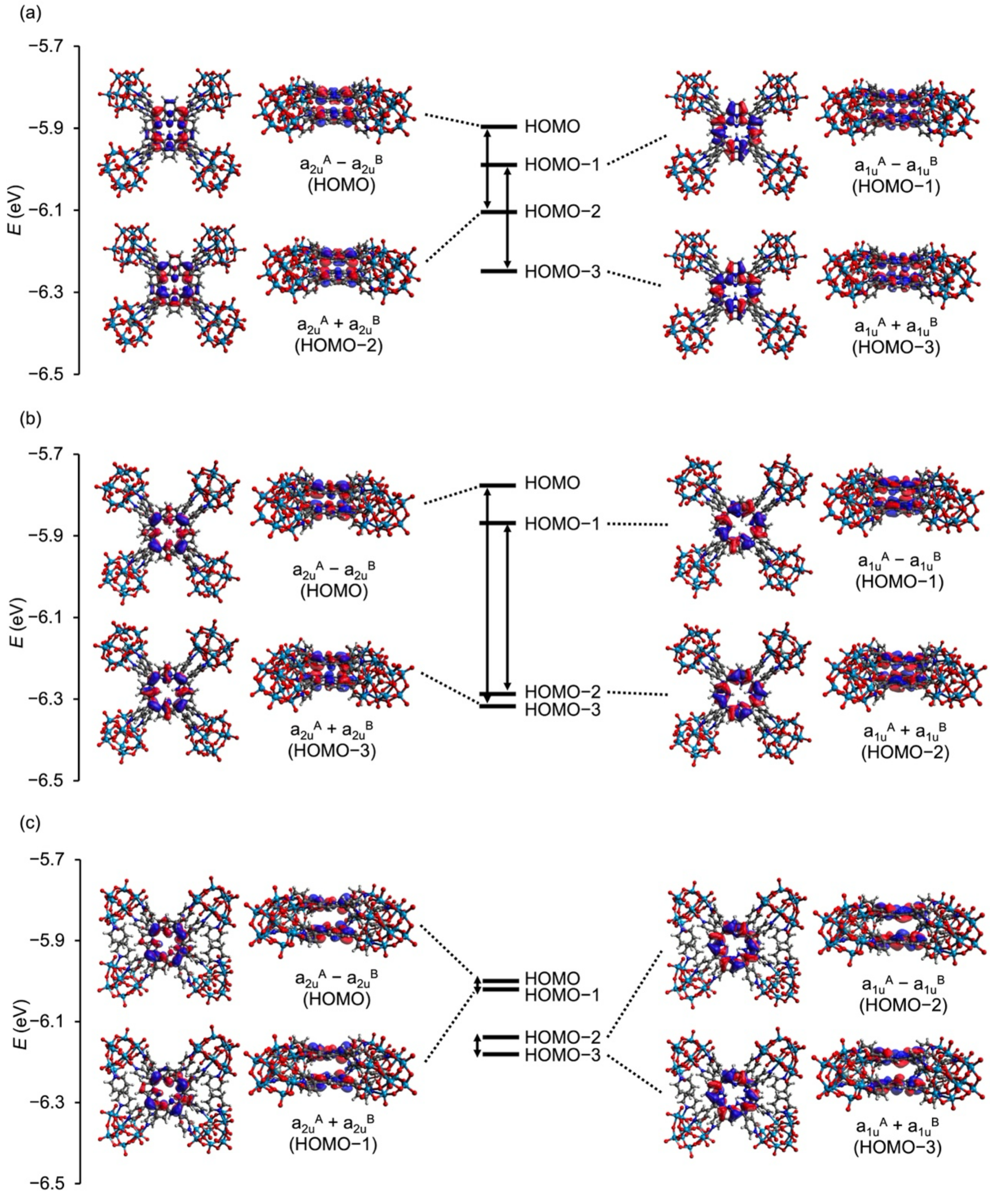

Fig. S5 DFT-calculated molecular orbitals (from HOMO − 3 to HOMO) of (a) hybrid **I**, (b) hybrid **II**, and (c) hybrid **III**. One porphyrin unit in the porphyrin dimer as A and the other porphyrin unit as B. The splitting of a_{1u} and a_{2u} orbitals is the interplanar resonance integrals strongly correlated with the orbital overlap between the porphyrins. The calculated splitting of a_{1u} and a_{2u} orbitals is in good agreement with the observed degree of redshift in the Q band.

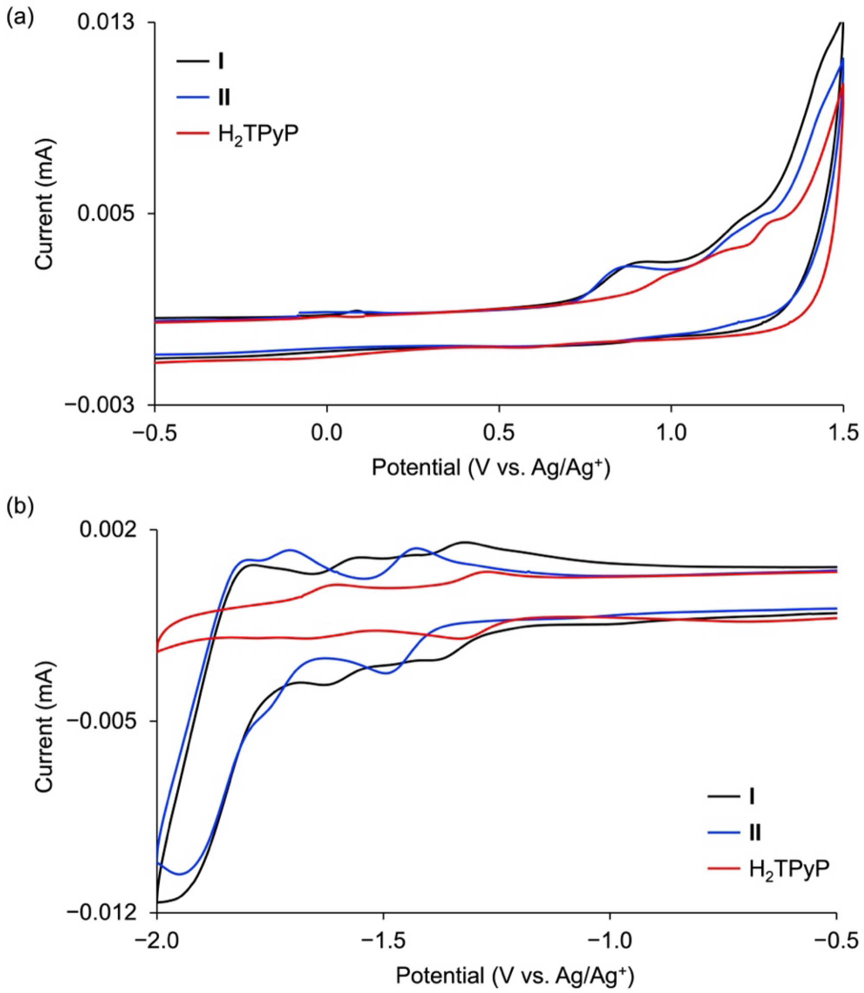

Fig. S6 Cyclic voltammogram of hybrids **I** and **II** and monomeric H_2TPyP in acetonitrile/dichloromethane (1/9, *v*/*v*) containing 0.1 M $TBAClO_4$ under Ar (1 atm) in the range of (a) −0.5 to 1.5 V vs. Ag/Ag^+ and (b) −2.0 to −0.5 V vs. Ag/Ag^+. The second cycles of the voltammogram were shown.

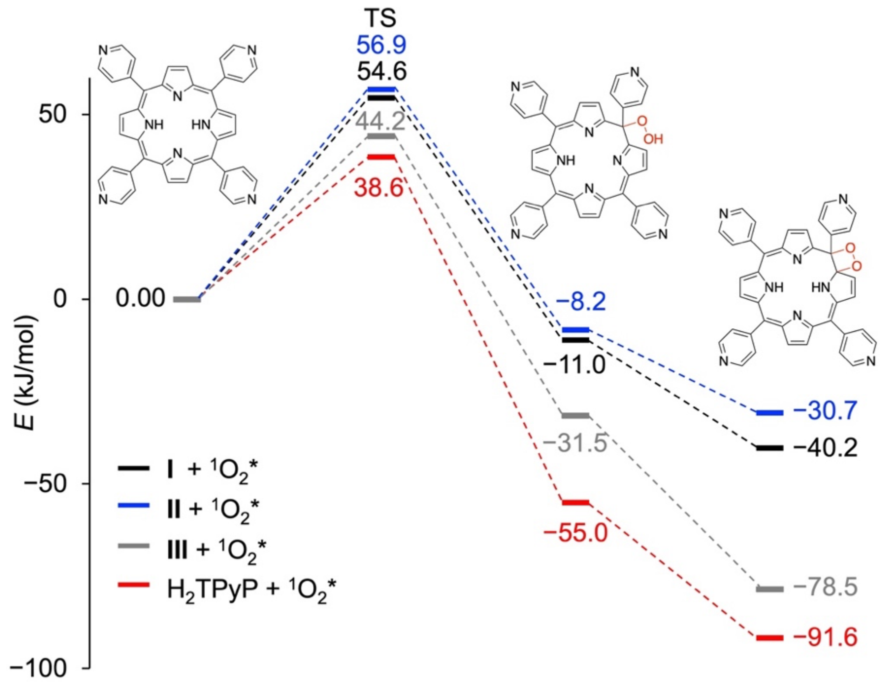

Fig. S7 DFT-calculated energy diagrams along decay process caused by addition of $^1O_2$* to hybrids **I**, **II**, **III**, and monomeric H_2TPyP.

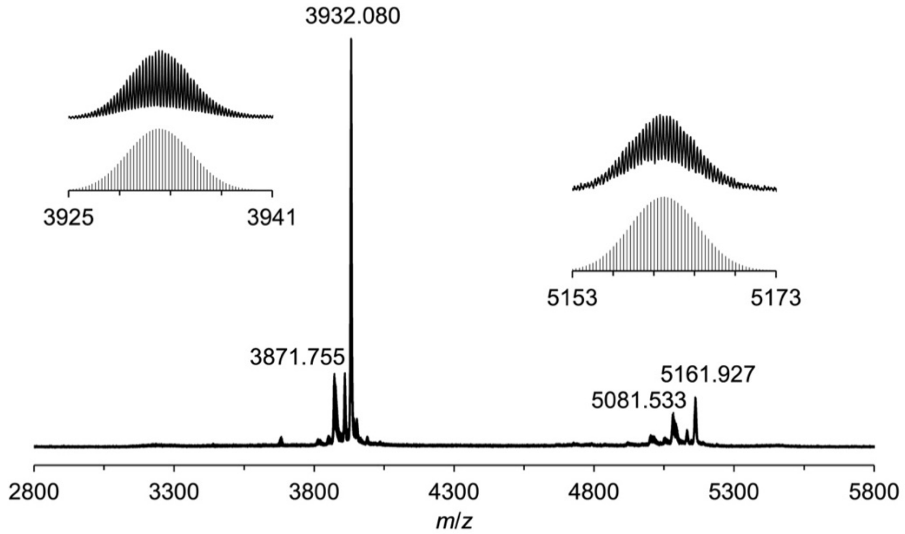

Fig. S8 ESI-mass spectrum of the retrieved **II** after the photo-oxidation of **1a** under visible-light irradiation. Insets: (top) observed spectra in *m/z* ranges of 3925−3941 and 5153−5173; (bottom) simulated patterns for $[TBA_{20}(SiW_{10}O_{34})_4(H_2TPyP)_2]^{4+}$ (theoretical *m/z* 3932.106) and $[TBA_{19}(SiW_{10}O_{34})_4(H_2TPyP)_2]^{3+}$ (theoretical *m/z* 5162.047). Signals attributed to $[TBA_{19}H(SiW_{10}O_{34})_4(H_2TPyP)_2]^{4+}$ (theoretical *m/z* 3871.787) and $[TBA_{18}H(SiW_{10}O_{34})_4(H_2TPyP)_2]^{3+}$ (theoretical *m/z* 5081.621) were also observed.

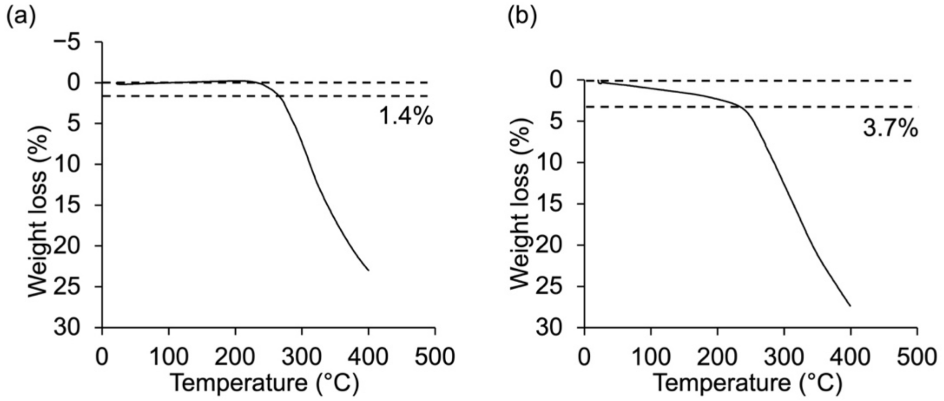

Fig. S9 TG-DTA of (a) hybrid **II** and (b) hybrid **III**.

References

S1 (a) K. Kamata, K. Yonehara, Y. Sumida, K. Yamaguchi, S. Hikichi and N. Mizuno, *Science*, 2003, **300**, 964; b) M. Yamaguchi, K. Shioya, C. Li, K. Yonesato, K. Murata, K. Ishii, K. Yamaguchi and K. Suzuki, *J. Am. Chem. Soc*., 2024, **146**, 4549.

S2 T. Minato, K. Suzuki, K. Yamaguchi and N. Mizuno, *Chem. Eur. J.*, 2017, **23**, 14213.

S3 Rigaku OD. CrysAlis PRO. Rigaku Oxford Diffraction Ltd, Yarnton, England (2018).

S4 (a) O. V. Dolomanov, L. J. Bourhis, R. J. Gildea, J. A. K. Howard and H. Puschmann, *J. Appl. Cryst.*, 2009. **42**, 339; (b) L. J. Farrugia, *J. Appl. Cryst.*, 2012, **45**, 849.

S5 G. M. Sheldrick, *Acta Cryst.*, 2015, **A71**, 3.

S6 (a) G. M. Sheldrick, *Acta Cryst.*, 2008, **A64**, 112; (b) G. M. Sheldrick, *Acta Cryst.*, 2015, **C71**, 3.

S7 P. van der Sluis and A. L. Spek, *Acta Cryst.*, 1990, **A46**, 194.

S8 N. E. Brese and M. O'Keeffe, *Acta Cryst.*, 1991, **B47**, 192.

S9 R. Schmidt, K. Seikel and H. D. Brauer, *J. Phys. Chem.*, 1989, **93**, 4507.

S10 S. Grimme, J. Antony, S. Ehrlich and H. Krieg, *J. Chem. Phys*., 2010, **132**, 154104.
